# Early Diagnosis of Pancreatic Ductal Adenocarcinoma by Combining Relative Expression Orderings With Machine-Learning Method

**DOI:** 10.3389/fcell.2020.582864

**Published:** 2020-10-15

**Authors:** Zi-Mei Zhang, Jia-Shu Wang, Hasan Zulfiqar, Hao Lv, Fu-Ying Dao, Hao Lin

**Affiliations:** Key Laboratory for Neuro-Information of Ministry of Education, Center for Informational Biology, School of Life Sciences and Technology, University of Electronic Science and Technology of China, Chengdu, China

**Keywords:** pancreatic ductal adenocarcinoma, biomarker, relative expression orderings, diagnosis, support vector machine

## Abstract

Pancreatic ductal adenocarcinoma (PDAC) is an aggressive and lethal cancer deeply affecting human health. Diagnosing early-stage PDAC is the key point to PDAC patients’ survival. However, the biomarkers for diagnosing early PDAC are inexact in most cases. Therefore, it is highly desirable to identify an effective PDAC diagnostic biomarker. In the current work, we designed a novel computational approach based on within-sample relative expression orderings (REOs). A feature selection technique called minimum redundancy maximum relevance was used to pick out optimal REOs. We then compared the performances of different classification algorithms for discriminating PDAC and its adjacent normal tissues from non−PDAC tissues. The support vector machine algorithm is the best one for identifying early PDAC diagnostic biomarker. At first, a signature composed of nine gene pairs was acquired from microarray gene expression data sets. These gene pairs could produce satisfactory classification accuracy up to 97.53% in fivefold cross-validation. Subsequently, two types of data from diverse platforms, namely, microarray and RNA-Seq, were used to validate this signature. For microarray data, all (100.00%) of 115 PDAC tissues and all (100.00%) of 31 PDAC adjacent normal tissues were correctly recognized as PDAC. In addition, 88.24% of 17 non-PDAC (normal or pancreatitis) tissues were correctly classified. For the RNA-Seq data, all (100.00%) of 177 PDAC tissues and all (100.00%) of 4 PDAC adjacent normal tissues were correctly recognized as PDAC. Validation results demonstrated that the signature had a good cross-platform effect for early detection of PDAC. This work developed a new robust signature that might be a promising biomarker for early PDAC diagnosis.

## Introduction

Pancreatic ductal adenocarcinoma (PDAC) is one of the deadliest malignant carcinomas and it accounts for at least 95% of all pancreatic cancer cases (Tanaka, 2016). PDAC has a poor survival outcome (Zhang et al., 2018b) by reason of the difficulty of diagnosing and assessing PDAC at an early stage. Most patients with PDAC do not present any specific early characteristics during the early stage, which means that early PDAC cannot be detected timely and thus causes missed chances for surgery. At present, the most commonly and widely used tumor biomarker for early PDAC diagnosis is carbohydrate antigen 19-9 (CA19-9) (Goggins, 2005), but it is not an ideal biomarker because of its relatively low level of sensitivity and specificity (70% with a 5% error rate, for diagnosis of PDAC) (Goonetilleke and Siriwardena, 2007; Datta and Vollmer, 2014). Therefore, a reliable signature with exquisitely high sensitivity and specificity is urgently needed to facilitate early PDAC diagnosis.

The main shortcoming of the existing diagnostic signatures is that they are basically obtained by using signature genes’ absolute expression value (Klett et al., 2018; Liao et al., 2018; Lu et al., 2018; Cheng et al., 2019b; Zou and Ma, 2020). Therefore, the batch effects could influence the choice of diagnostic signatures. Luckily, we could obtain diagnostic signatures with qualitative transcriptional information through exploiting the relative expression ordering (REO) method. The REO method is highly robust to experimental batch effects (Eddy et al., 2010; Cai et al., 2015; Zhao et al., 2016) and platform differences (Guan et al., 2016; Cheng, 2019). Therefore, it is possible to find robust and reliable disease signatures by using the datasets integrated from different platforms. Moreover, the REO strategy has been successfully used to identify the early diagnosis signature of malignant carcinoma, such as gastric cancer (Yan et al., 2019), hepatocellular carcinoma (Ao et al., 2018), and colorectal cancer (Guan et al., 2019). Consequently, it is worth employing the within-sample REO method to develop a robust qualitative signature for diagnosing early-stage PDAC.

Machine-learning techniques, which can be used to uncover biological principles and mechanism, is a good choice for biological knowledge mining (Liu et al., 2013, 2019; Cao et al., 2017; Cheng et al., 2018a,b; Du et al., 2018; Zou et al., 2018; Stephenson et al., 2019). Hence, this work was devoted to develop an artificial intelligence-based approach to identify early-stage PDAC diagnostic signature. In the first step, all REOs were used for initial diagnosis descriptor. Subsequently, the minimum redundancy maximum relevance (mRMR), a features selection technique, was utilized to remove redundant REOs. The support vector machine (SVM), decision tree, logistic regression, random forest, naïve Bayes, and Bayes net algorithms were used for classification. Finally, 9 salient and genuine gene pairs including 16 genes were screened as the diagnostic signature for diagnosing early-stage PDAC. The nine gene pairs’ signature displayed good diagnosis performance for early-stage PDAC in different diagnosis platforms by combining with SVM.

## Materials and Methods

### The Construction of Datasets

The microarray gene expression data and RNA-seq data used in current paper were collected from two databases: the GEO database^1^ and TCGA database^2^. The detailed description of all data sets is elucidated in Table 1.

**TABLE 1:** Statistics of all data sets.

Data set	Platform	PDAC	PDAC_adjacent	Pancreatitis	Normal
GSE62452	Affymetrix GPL6244	69	61	–	–
GSE28735	Affymetrix GPL6244	45	45	–	–
GSE22780	Affymetrix GPL570	8	8	–	–
GSE15471	Affymetrix GPL570	39	39	–	–
GSE50827	Illumina GPL10558	103	–	–	–
GSE106189	Affymetrix GPL570	35	–	–	–
GSE84219	Illumina GPL14951	30	–	–	–
GSE98399	Affymetrix GPL570	43	–	–	–
GSE62165	Affymetrix GPL13667	118	–	–	13
GSE32676	Affymetrix GPL570	25	–	–	7
GSE101462	Illumina GPL10558	6	–	10	4
GSE101448	Illumina GPL10558	24	–	–	19
GSE41368	Affymetrix GPL6244	6	–	–	6
GSE60601	Affymetrix GPL570	9	–	–	3
GSE71989	Affymetrix GPL570	13	–	–	8
GSE89120	Affymetrix GPL1352	–	–	–	14
Total		573	153	10	74
Samples for assessing the efficiency of the signature					
TCGA	RNA-Seq	177	4	–	–
Total		177	4		

*PDAC, pancreatic ductal adenocarcinoma tissues; PDAC_adjacent, PDAC adjacent normal tissues.*

Microarray data performed by the platform of Affymetrix and Illumina were freely downloaded from the GEO database. It contained 573 PDAC samples (Set1), 153 PDAC adjacent normal samples (Set2), 10 pancreatitis samples (Set3), and 74 normal samples (Set4). For data performed by Affymetrix, the raw data were directly downloaded from GEO and then the robust multi-array averaging (Bolstad et al., 2003; Irizarry et al., 2003a,b) was used to do the background correction and normalization. For data performed by Illumina, the originally processed data (series matrix files) were used. For all microarray data, the mapping information of probe IDs and Entrez gene IDs can be found in the corresponding platform files. For one gene with multiple probes, we used the arithmetic mean of these probes’ values as this gene’s expression value.

The RNA-Seq data set included 177 PDAC and 4 adjacent normal samples. We downloaded the free RNA-Seq profiles from TCGA (up to November 19, 2019) website using the TCGAbiolinks R package (Colaprico et al., 2016). The gene symbol expression matrix was obtained by using Ensembl gene IDs.

To make the evaluation of the model more objective, each category of samples (Set1, Set2, Set3, Set4) were divided into two subsets of data: training set (80% of each category of samples) and testing set (20% of each category of samples). Ultimately, the training set contained 580 tumor samples (458 PDAC samples and 122 PDAC adjacent normal samples) and 67 non-tumor samples (59 normal samples and 8 pancreatitis samples). The testing set contained 146 tumor samples (115 PDAC samples and 31 PDAC adjacent normal samples) and 17 non-tumor samples (15 normal samples and 2 pancreatitis samples) for the performance evaluation of the signature. In addition, the RNA-Seq data set and testing set belong to the independent test data sets.

### Relative Expression Orderings (REOs)

To obtain a more robust and reliable signature from gene expression profiles, REO methodology was utilized for feature construction. The REO for gene pair (i and j) is formulated as Gi > Gj or Gi < Gj, where Gi and Gj represent the expression values of gene i and j. For the gene pair, if more than 85% of the samples have the same REO, we deem this REO as a stable REO of this gene pair. The stable reversal gene pairs represent the gene pairs that have stable REOs in both tumor tissues and control tissues, but REO patterns are different (Gi > Gj or Gi < Gj in tumor tissues but Gi > Gj or Gi < Gj in non-tumor tissues). Also, the reversal stable gene pairs between tumor and control samples were chosen as the candidate REO-based qualitative diagnostic signature. We later gained the consistent genes of all preprocessed data sets and its corresponding gene expression profiles. Whereafter, based on the reversal gene pairs and gene expression profiles, we gained new profiles by using 0, 1, and −1 to denote Gi > Gj, Gi < Gj, and other cases (Gi or Gj does not exist), respectively.

### Minimum Redundancy Maximum Relevance (mRMR)

The mRMR (Peng et al., 2005) approach can omit the redundant features and choose the high-relevancy features to the target class, and thus significantly improve the classification accuracy. mRMR is on the base of information theory and it can be accomplished through mutual information (MI) operation, and the MI is formulated as follows:

(1) $$MI\left(v_{i},C\right)=\int p\left(v_{i},C\right)\ln\left(\frac{p\left(v_{i},C\right)}{p\left(v_{i}\right)p\left(C\right)}\right){\mathrm{dv}}_{i}\mathrm{dC}$$

where *v* represents the feature vector and *C* represents the class to be targeted.

The mRMR is estimated as

(2) $$mRMR=\frac{1}{\left|\Psi \right|}\sum_{v_{i}\in \Psi }MI\left(v_{i},C\right)-\frac{1}{{\left|\Psi \right|}^{2}}\sum_{v_{i}v_{j}\in \Psi }MI\left(v_{i},v_{j}\right)$$

where Ψ denotes the set of ranked features, MI (*v*_*i*_, *C*) denotes mutual information between the *v*_*i*_ feature and class C, and IM (*v*_*i*_,*v*_*j*_) denotes mutual information between *v*_*i*_ and *v*_*j*_.

In this work, a reversal stable gene pair was considered as a feature. The feature selection process was essential for exact classification between tumor samples (positive samples) and non-tumor samples (negative samples). Thus, we utilized mRMR method to pick out effective features (gene pairs).

### Incremental Feature Selection (IFS)

Based on mRMR techniques, we gained a list of ranked features (gene pairs). The incremental feature selection (IFS) (Li et al., 2019) strategy was adopted to find the optimal feature subset which could produce the best diagnosis for PDAC. During IFS process, the gene pair was added one by one to feature subset and the optimal features (gene pairs) were determined when the highest accuracy was obtained.

### Classification Algorithms

As a popular supervised learning approach, SVM was first introduced by Vapnik and has been widely used in various bioinformatics classification problems (Song et al., 2009; Shoombuatong et al., 2012; Win et al., 2017, 2018; Chen et al., 2019b; Laengsri et al., 2019; Manavalan et al., 2019b; Schaduangrat et al., 2019; Hasan et al., 2020; Liu and Chen, 2020). Herein, the free LibSVM (version 3.23) package (Chang and Lin, 2011) was employed to execute SVM. The LibSVM with fivefold cross-validation and radial basis function was employed to perform classification. The grid search with fivefold cross-validation was used to determine the *C* and *γ* values for SVM. As a result, we obtained the optimal values 32 and 0.03125 for *C* and *γ*, respectively. Apart from SVM, decision tree, logistic regression, random forest, naïve Bayes, and Bayes net were also utilized as classification algorithm and performed by using Weka (version 3.8.3) (Frank et al., 2004). Within this research, the aforementioned six classification algorithms with fivefold cross-validation were used.

### Performance Measurements

In the current paper, six indexes were used to measure the effectiveness of our model. They are accuracy (ACR), sensitivity (SES), specificity (SPF), Matthews correlation coefficient (MCC) (Li et al., 2015; Bao et al., 2019; Chen et al., 2019a; Cheng et al., 2019a, 2020), the receiver operating characteristic (ROC) curves, and the area under the ROC curve (AUC). Especially, taking into consideration the class imbalance of tumor tissues and non-tumor tissues, we appointed MCC as the major performance measurement in this work. The details about ACR, SES, SPF, and MCC can be found from Tang et al. (2017); Basith et al. (2019), Manavalan et al. (2019a); Patil and Chouhan (2019), and Basith et al. (2020).

## Results

### Derivation of PDAC Diagnostic Signature

**FIGURE 1:**
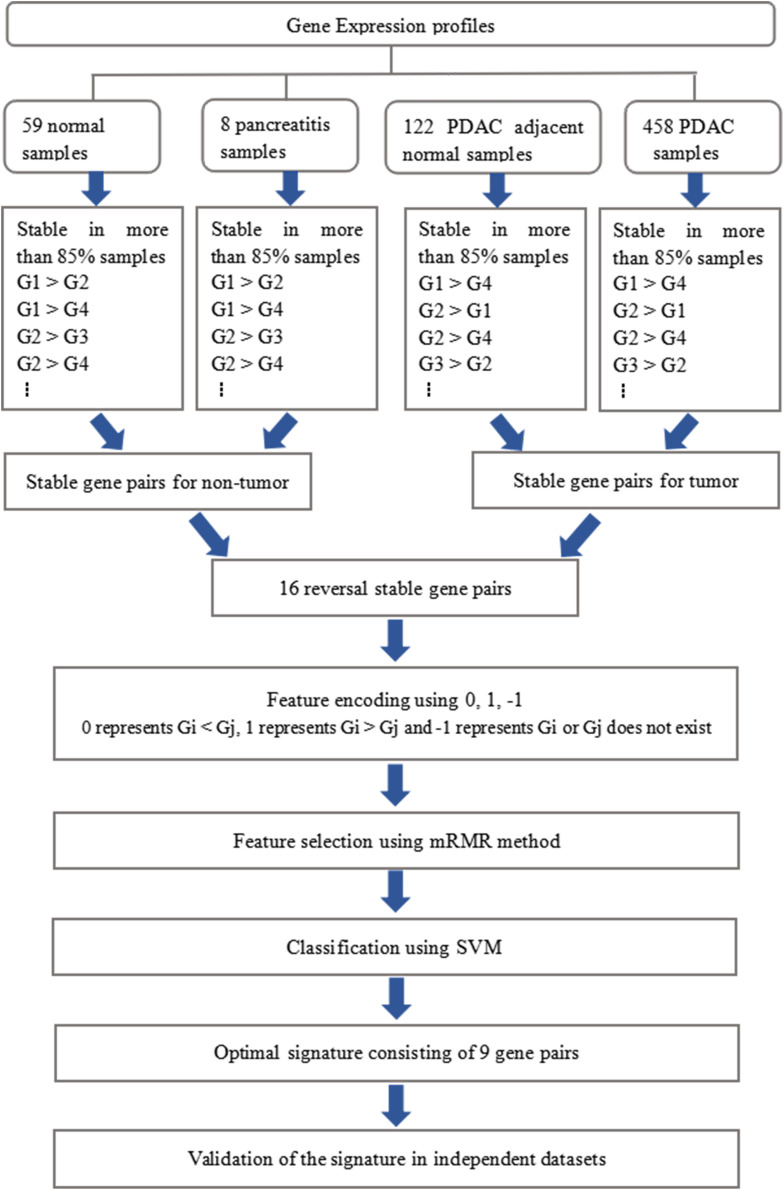
Schematic workflow of analyses.

**FIGURE 2:**
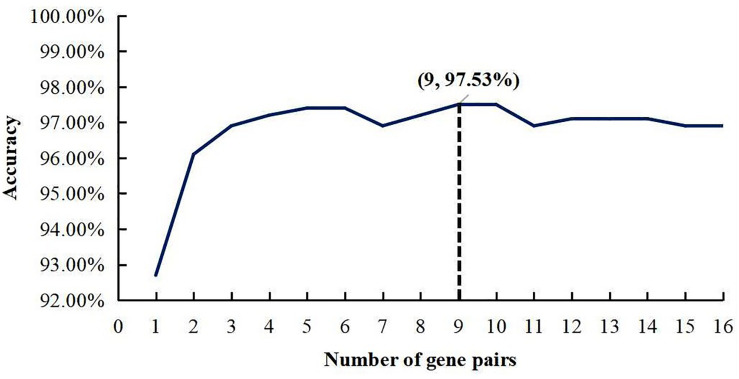
A plot to show the IFS curve. The black dotted line showed that nine gene pairs reached the highest accuracy of 97.53%.

The whole procedure of deriving the diagnostic signature is provided in Figure 1. First, with the relative expression orderings elaborated in Materials and Methods section, for 458 PDAC samples and 122 PDAC adjacent normal samples in the training set, there were 30,865,512 and 49,177,748 stable gene pairs, respectively. Also, there were 17,842,291 consistent stable gene pairs in total. Likewise, for 8 pancreatitis samples and 59 normal samples in the training set, there were 53,719,117 and 44,523,890 stable gene pairs, respectively. There were 25,687,362 consistent stable gene pairs in total. Among 17,842,291 and 25,687,362 gene pairs, there were 16 stable reversal gene pairs between the two sets of samples. Then, on the basis of the novel profiles (see Materials and Methods), we captured the optimal feature set from the 16 gene pairs by using mRMR with SVM, decision tree, logistic regression, random forest, naïve Bayes, and Bayes net. The comparison results of the aforementioned six classification algorithms are listed in Table 2. It was obvious that the SVM algorithm was the best one for identifying early PDAC diagnostic biomarker. The accuracy, sensitivity, specificity, and MCC of SVM were, respectively, 97.53%, 97.96%, 93.22% and 0.8615. Therefore, the final model used for early PDAC diagnostic biomarker identification was built based on SVM algorithm. The blue curve in Figure 2 displayed the process of IFS method. As we could see from Figure 2, with fivefold cross-validation, the nine gene pair signature could identify PDAC with up to 97.53% accuracy on training set. That is to say, nine gene pairs illustrated in Table 3 were deemed as the optimal signature for diagnosing the early-stage PDAC.

**TABLE 2:** Comparison of different methods for identifying early PDAC diagnostic biomarker.

Methods	Training set				Testing set			
	ACR (%)	SES (%)	SPF (%)	MCC	ACR (%)	SES (%)	SPF (%)	MCC
SVM	97.53	97.96	93.22	0.8615	98.77	98.65	100.00	0.9330
Decision tree	96.91	97.78	88.52	0.8278	95.09	97.92	73.68	0.7518
Logistic regression	96.91	98.11	86.15	0.8314	96.93	99.30	80.00	0.8513
Random forest	96.60	97.61	86.89	0.8104	96.93	99.30	80.00	0.8513
Naïve Bayes	96.14	98.94	76.25	0.8124	96.32	99.30	76.19	0.8274
Bayes net	95.83	98.59	75.64	0.7933	95.70	99.29	72.73	0.8051

*PDAC, pancreatic ductal adenocarcinoma tissues; PDAC_adjacent, PDAC adjacent normal tissues; ACR, accuracy; SES, sensitivity; SPF, specificity; MCC, Matthews correlation coefficient.*

**TABLE 3:** The nine gene pairs’ signature ranked by mRMR.

Order	Feature (gene pair)	
	Gene i	Gene j
1	UBE2C	FITM1
2	SERPINB5	ZNF100
3	NUSAP1	ONECUT1
4	LAMC2	RBM33
5	BCAR3	FBXO42
6	CTSE	PRRC2C
7	HOXB7	MYO19
8	NUSAP1	TNKS
9	RRM2	ONECUT1

*The absolute expression value of gene i is higher than that of gene j in PDAC patients compared with non-PDAC patients.*

### Examination of the Signature

**FIGURE 3:**
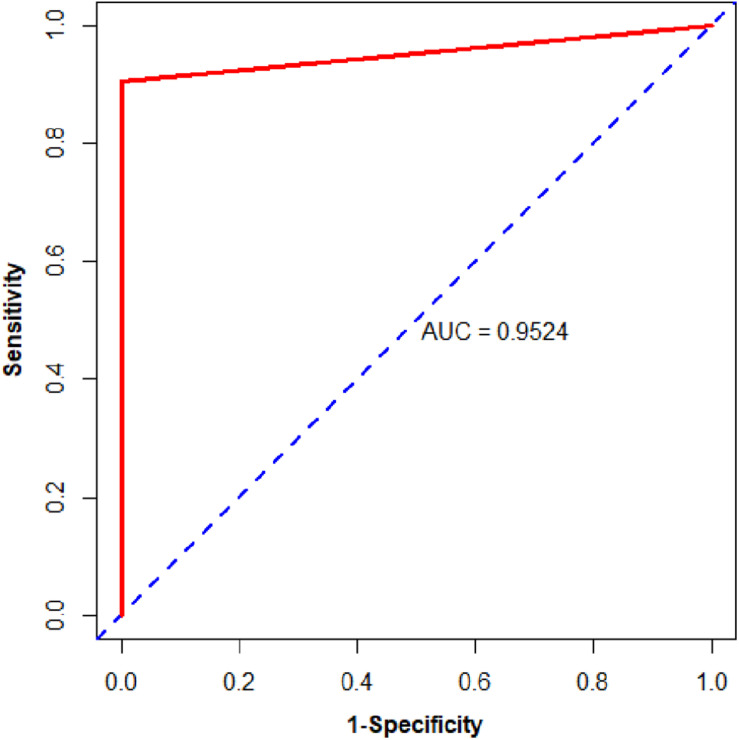
The ROC curve of the independent test data sets.

We then assessed the classification ability of nine gene pairs in independent test data sets, and the test results with fivefold cross-validation are shown in Table 4. For 163 samples in the testing set, our model reached accuracy, sensitivity, specificity, and MCC values of 98.77%, 98.65%, 100.00%, and 0.9330, respectively. Furthermore, the signature 9 gene pairs could accurately distinguish 177 PDAC samples and 4 PDAC adjacent normal samples measured by RNA-Seq although the training set did not contain any RNA-Seq information. This test result, based on RNA-Seq data set, indicated that the nine gene pairs have a good cross-platform effect for PDAC early detection. For all 327 PDAC samples and 17 non-PDAC samples collected from public databases, the accuracy, sensitivity, specificity, and MCC are 99.42%, 99.39%, 100%, and 0.9365, respectively. Also, the AUC reached 0.9524 (95% CI, 0.8881–1; see Figure 3). According to independent tests on testing set and RNA-Seq data set, it was concluded that the signature could discriminate PDAC (PDAC and adjacent normal tissues) patients from non-PDAC (pancreatitis and normal tissues) patients.

**TABLE 4:** Classification efficiency of the nine gene pairs in independent test data sets.

Data set	PDAC	PDAC_adjacent	Pancreatitis	Normal	ACR	SES	SPF	MCC
Testing set	115	31	2	15	98.77%	98.65%	100.00%	0.9330
TCGA	177	4	–	–	–	100.00%	–	–

*PDAC, pancreatic ductal adenocarcinoma tissues; PDAC_adjacent, PDAC adjacent normal tissues; ACR, accuracy; SES, sensitivity; SPF, specificity; MCC, Matthews correlation coefficient.*

## Discussion

Pancreatic carcinoma is a life-threatening malignant tumor of the digestive system with bad prognosis due to late diagnosis. The current imaging techniques and existing tumor signatures have insufficient sensitivity and/or specificity for early PDAC diagnosis. Herein, new strategies for diagnosis at an early stage of the disease are urgently needed. In the current work, we found a robust qualitative diagnostic signature 9 gene pairs (16 genes), which can discriminate PDAC (PDAC and adjacent normal tissues) patients from non-PDAC (pancreatitis and normal tissues) and might be a promising biomarker for early diagnosis of PDAC.

Database PubMed was searched and retrieved appropriate journal articles on the association between 16 genes in 9 gene pairs and PDAC published before August 18, 2020. Seven genes in the nine gene pairs’ signature, including UBE2C, SERPINB5, LAMC2, CTSE, HOXB7, RRM2, and ONECUT1, had been reported to be related to PDAC. The description of the association between seven genes and PDAC is displayed in Table 5. They might play a vital role in PDAC tumorigenesis and were critical genes for cancer. Notably, CTSE (Keliher et al., 2013), HOXB7 (Chile et al., 2013; Nguyen Kovochich et al., 2013), and RRM2 (Bhutia et al., 2013) were overexpressed in PDAC. UBE2C could encode a ubiquitin-conjugating enzyme which correlated with the PDAC development and progression. Also, the proliferation and epithelial–mesenchymal transition in PDAC could be inhibited by silencing UBE2C (Wang et al., 2019). SERPINB5 had been found to link to the prognosis of PDAC (Cheng et al., 2019b). LAMC2 has relation with the occurrence and progression of PDAC patients (Pan et al., 2018; Yang et al., 2018; Zhang et al., 2018a). Furthermore, the high expression level of LAMC2 could facilitate the invasion of PDAC cell and thus increase the risk of tumor recurrence (Yang et al., 2018). Patients with pancreatic diseases (chronic pancreatitis) had a higher risk of developing PDAC and thus the expression of CTSE in pancreatic diseases might be the key to early PDAC detection and PDAC progression. HOXB7, frequently overexpressed in PDAC, closely connected with lymph node metastasis (Nguyen Kovochich et al., 2013) and worse survival in PDAC patients (Zhang et al., 2014). Knockdowning HOXB7 could cause cell apoptosis and cell cycle arrest (Chile et al., 2013). RRM2 was involved in the process of deoxyribonucleotide synthesis. Gene expression of RRM2 was significantly higher in PDAC tissues than in normal pancreatic tissues, which brought about the chemoresistance of PDAC to nucleoside analogs (Bhutia et al., 2013). A loss of ONECUT1 expression in PDAC cells implied its tumor suppressor function in this malignant tumor (Jiang et al., 2008).

**TABLE 5:** The description of the association between seven genes and PDAC.

Gene symbol	The description of the association between seven genes and PDAC
UBE2C	Silencing UBE2C could inhibit the proliferation and epithelial–mesenchymal transition in PDAC (Manavalan et al., 2019a)
SERPINB5	SERPINB5 links to the prognosis of PDAC (Cheng et al., 2019b)
LAMC2	LAMC2 is associated with PDAC occurrence and progression (54–56). The high expression level of LAMC2 could facilitate the invasion of PDAC cell and thus increase the risk of tumor recurrence (Keliher et al., 2013)
CTSE	Because patients with pancreatic diseases (chronic pancreatitis) have a strong risk of developing PDAC, the expression of CTSE in pancreatic diseases might be the key to detection of early PDAC and progression of PDAC
HOXB7	HOXB7 is overexpressed in PDAC. It is closely relevant to lymph node metastasis (Patil and Chouhan, 2019) and worse survival of PDAC patients (Chile et al., 2013). Knockdowning HOXB7 can cause cell apoptosis and cell cycle arrest (Tang et al., 2017)
RRM2	Gene expression of RRM2 was significantly higher in PDAC tissues than normal pancreatic tissues (Basith et al., 2019)
ONECUT1	Loss expression of ONECUT1 in PDAC cells implied its tumor suppressor function in this malignant tumor (Wang et al., 2019)

To further study the detailed information and functions of the 9 gene pairs, we analyzed 16 genes (9 gene pairs) via using online tools in Metascape^3^ (Tripathi et al., 2015). The enrichment analysis included GO terms functional enrichment and KEGG pathway enrichment. Pathways with *P*-value were less than 0.05 and the number of enriched genes greater than or equal to 3 was considered significant. Ultimately, based on the GO enrichment, the 16 genes (9 gene pairs) enriched in two terms in the category BP, including “regulation of cell cycle process” and “regulation of mitotic nuclear division.” UBE2C, RRM2, TNKS, NUSAP1, and MYO19 were included in the genes enriched in regulation of cell cycle process, whereas TNKS, UBE2C, NUSAP1, and MYO19 enriched in the regulation of mitotic nuclear division. Collecting the aforementioned results, the genes of the nine gene pairs might play a significant part in the tumorigenesis of PDAC.

In conclusion, we had identified nine gene pairs’ signature for early-stage PDAC diagnosis that could correctly distinguish PDAC (PDAC and PDAC adjacent normal tissues) tissues from non−PDAC (normal and pancreatitis tissues) patients at individual level. Because the number of normal and pancreatitis samples used in the current work for distinguishing early-stage PDAC is relatively small, we will try to collect more samples from more public databases to further obtain a novel diagnostic signature with higher accuracy on larger numbers of such specimens. Moreover, we hope that some RNA signature (Fang et al., 2019; Vaschetto, 2019; Wu et al., 2019) can be found and applied in related fields.

## Data Availability Statement

Publicly available datasets were analyzed in this study. This data can be found here: https://portal.gdc.cancer.gov/ and http://www.ncbi.nlm.nih.gov/geo/.

## Author Contributions

HL designed and supervised the study. Z-MZ collected all datasets and wrote the article with the help of HL and F-YD. Z-MZ, J-SW, HZ, HL, and F-YD performed the experiments. All authors contributed to the article and approved the submitted version.

## Conflict of Interest

The authors declare that the research was conducted in the absence of any commercial or financial relationships that could be construed as a potential conflict of interest.

## Footnotes

1 http://www.ncbi.nlm.nih.gov/geo/

2 https://portal.gdc.cancer.gov/

3 http://metascape.org

## Abbreviations

PDAC: pancreatic ductal adenocarcinoma
REOs: relative expression orderings
mRMR: maximum relevance minimum redundancy
IFS: incremental feature selection
SVM: support vector machine.

## References

[B1] Ao L. Zhang Z. Guan Q. Guo Y. Guo Y. Zhang J. (2018). A qualitative signature for early diagnosis of hepatocellular carcinoma based on relative expression orderings. *Liver Int.* 38 1812–1819. 10.1111/liv.13864 29682909 PMC6175149

[B2] Bao Y. Marini S. Tamura T. Kamada M. Maegawa S. Hosokawa H. (2019). Toward more accurate prediction of caspase cleavage sites: a comprehensive review of current methods, tools and features. *Brief. Bioinform.* 20 1669–1684. 10.1093/bib/bby041 29860277 PMC6917222

[B3] Basith S. Manavalan B. Shin T. H. Lee G. (2019). SDM6A: a web-based integrative machine-learning framework for predicting 6mA sites in the rice genome. *Mol. Ther. Nucleic Acids* 18 131–141. 10.1016/j.omtn.2019.08.011 31542696 PMC6796762

[B4] Basith S. Manavalan B. Shin T. H. Lee G. (2020). Machine intelligence in peptide therapeutics: a next-generation tool for rapid disease screening. *Med. Res. Rev.* 40 1276–1314. 10.1002/med.21658 31922268

[B5] Bhutia Y. D. Hung S. W. Krentz M. Patel D. Lovin D. Manoharan R. (2013). Differential processing of let-7a precursors influences RRM2 expression and chemosensitivity in pancreatic cancer: role of LIN-28 and SET oncoprotein. *PLoS One* 8:e53436. 10.1371/journal.pone.0053436 23335963 PMC3546076

[B6] Bolstad B. M. Irizarry R. A. Astrand M. Speed T. P. (2003). A comparison of normalization methods for high density oligonucleotide array data based on variance and bias. *Bioinformatics* 19 185–193. 10.1093/bioinformatics/19.2.185 12538238

[B7] Cai H. Li X. Li J. Ao L. Yan H. Tong M. (2015). Tamoxifen therapy benefit predictive signature coupled with prognostic signature of post-operative recurrent risk for early stage ER+ breast cancer. *Oncotarget* 6 44593–44608. 10.18632/oncotarget.6260 26527319 PMC4792578

[B8] Cao R. Freitas C. Chan L. Sun M. Jiang H. Chen Z. (2017). ProLanGO: protein function prediction using neural machine translation based on a recurrent neural network. *Molecules* 22:1732. 10.3390/molecules22101732 29039790 PMC6151571

[B9] Chang C. C. Lin C. J. (2011). LIBSVM: a library for support vector machines. *ACM T Intel. Syst. Tec.* 2 1–27. 10.1145/1961189.1961199

[B10] Chen W. Feng P. Liu T. Jin D. (2019a). Recent advances in machine learning methods for predicting heat shock proteins. *Curr. Drug Metab* 20 224–228. 10.2174/1389200219666181031105916 30378494

[B11] Chen W. Feng P. Nie F. (2019b). iATP: a sequence based method for identifying anti-tubercular peptides. *Med. Chem.* 16 620–625. 10.2174/1573406415666191002152441 31339073

[B12] Cheng L. (2019). Computational and biological methods for gene therapy. *Curr. Gene Ther.* 19:210. 10.2174/156652321904191022113307 31762421

[B13] Cheng L. Hu Y. Sun J. Zhou M. Jiang Q. (2018a). DincRNA: a comprehensive web-based bioinformatics toolkit for exploring disease associations and ncRNA function. *Bioinformatics* 34 1953–1956. 10.1093/bioinformatics/bty002 29365045

[B14] Cheng L. Jiang Y. Ju H. Sun J. Peng J. Zhou M. (2018b). InfAcrOnt: calculating cross-ontology term similarities using information flow by a random walk. *BMC Genomics* 19:919. 10.1186/s12864-017-4338-6 29363423 PMC5780854

[B15] Cheng L. Qi C. Zhuang H. Fu T. Zhang X. (2020). gutMDisorder: a comprehensive database for dysbiosis of the gut microbiota in disorders and interventions. *Nucleic Acids Res.* 48 D554–D560. 31584099 10.1093/nar/gkz843 PMC6943049

[B16] Cheng L. Zhao H. Wang P. Zhou W. Luo M. Li T. (2019a). Computational methods for identifying similar diseases. *Mol. Ther. Nucleic Acids* 18 590–604. 10.1016/j.omtn.2019.09.019 31678735 PMC6838934

[B17] Cheng Y. Wang K. Geng L. Sun J. Xu W. Liu D. (2019b). Identification of candidate diagnostic and prognostic biomarkers for pancreatic carcinoma. *EBIO Med.* 40 382–393. 10.1016/j.ebiom.2019.01.003 30639415 PMC6412825

[B18] Chile T. Fortes M. A. Correa-Giannella M. L. Brentani H. P. Maria D. A. Puga R. D. (2013). HOXB7 mRNA is overexpressed in pancreatic ductal adenocarcinomas and its knockdown induces cell cycle arrest and apoptosis. *BMC Cancer* 13:451. 10.1186/1471-2407-13-451 24088503 PMC3851693

[B19] Colaprico A. Silva T. C. Olsen C. Garofano L. Cava C. Garolini D. (2016). TCGAbiolinks: an R/Bioconductor package for integrative analysis of TCGA data. *Nucleic Acids Res.* 44:e71. 10.1093/nar/gkv1507 26704973 PMC4856967

[B20] Datta J. Vollmer C. M. Jr. (2014). Investigational biomarkers for pancreatic adenocarcinoma: where do we stand? *Southern Med. J.* 107 256–263. 10.1097/smj.0000000000000088 24937521

[B21] Du X. Q. Li X. R. Li W. Yan Y. T. Zhang Y. P. (2018). Identification and analysis of cancer diagnosis using probabilistic classification vector machines with feature selection. *Curr. Bioinform.* 13 625–632. 10.2174/1574893612666170405125637

[B22] Eddy J. A. Sung J. Geman D. Price N. D. (2010). Relative expression analysis for molecular cancer diagnosis and prognosis. *Technol. Cancer Res. Treatment* 9 149–159. 10.1177/153303461000900204 20218737 PMC2921829

[B23] Fang S. Pan J. C. Zhou C. W. Tian H. He J. X. Shen W. Y. (2019). Circular RNAs serve as novel biomarkers and therapeutic targets in cancers. *Curr. Gene Ther.* 19 125–133. 10.2174/1566523218666181109142756 30411680

[B24] Frank E. Hall M. Trigg L. Holmes G. Witten I. H. (2004). Data mining in bioinformatics using weka. *Bioinformatics* 20 2479–2481. 10.1093/bioinformatics/bth261 15073010

[B25] Goggins M. (2005). Molecular markers of early pancreatic cancer. *J. Clin. Oncol.* 23 4524–4531. 10.1200/jco.2005.19.711 16002843

[B26] Goonetilleke K. S. Siriwardena A. K. (2007). Systematic review of carbohydrate antigen (CA 19-9) as a biochemical marker in the diagnosis of pancreatic cancer. *Eur. J. Surg.* 33 266–270. 10.1016/j.ejso.2006.10.004 17097848

[B27] Guan Q. Chen R. Yan H. Cai H. Guo Y. Li M. (2016). Differential expression analysis for individual cancer samples based on robust within-sample relative gene expression orderings across multiple profiling platforms. *Oncotarget* 7 68909–68920. 10.18632/oncotarget.11996 27634898 PMC5356599

[B28] Guan Q. Zeng Q. Yan H. Xie J. Cheng J. Ao L. (2019). A qualitative transcriptional signature for the early diagnosis of colorectal cancer. *Cancer Sci.* 110 3225–3234. 10.1111/cas.14137 31335996 PMC6778657

[B29] Hasan M. M. Schaduangrat N. Basith S. Lee G. Shoombuatong W. Manavalan B. (2020). HLPpred-Fuse: improved and robust prediction of hemolytic peptide and its activity by fusing multiple feature representation. *Bioinformatics* 36 3350–3356. 10.1093/bioinformatics/btaa160 32145017

[B30] Irizarry R. A. Bolstad B. M. Collin F. Cope L. M. Hobbs B. Speed T. P. (2003a). Summaries of affymetrix genechip probe level data. *Nucleic Acids Res.* 31:e15. 10.1093/nar/gng015 PMC150247 12582260

[B31] Irizarry R. A. Hobbs B. Collin F. Beazer-Barclay Y. D. Antonellis K. J. Scherf U. (2003b). Exploration, normalization, and summaries of high density oligonucleotide array probe level data. *Biostatistics* 4 249–264. 10.1093/biostatistics/4.2.249 12925520

[B32] Jiang X. Zhang W. Kayed H. Zheng P. Giese N. A. Friess H. (2008). Loss of ONECUT1 expression in human pancreatic cancer cells. *Oncol. Rep.* 19 157–163. 18097590

[B33] Keliher E. J. Reiner T. Earley S. Klubnick J. Tassa C. Lee A. J. (2013). Targeting cathepsin E in pancreatic cancer by a small molecule allows in vivo detection. *Neoplasia* 15 684–693. 10.1593/neo.13276 23814481 PMC3689232

[B34] Klett H. Fuellgraf H. Levit-Zerdoun E. Hussung S. Kowar S. Kusters S. (2018). Identification and validation of a diagnostic and prognostic multi-gene biomarker panel for pancreatic ductal adenocarcinoma. *Front. Genet.* 9:108. 10.3389/fgene.2018.00108 29675033 PMC5895731

[B35] Laengsri V. Nantasenamat C. Schaduangrat N. Nuchnoi P. Prachayasittikul V. Shoombuatong W. (2019). TargetAntiAngio: a sequence-based tool for the prediction and analysis of anti-angiogenic peptides. *Int. J. Mol. Sci.* 20:2950. 10.3390/ijms20122950 31212918 PMC6628072

[B36] Li F. Li C. Wang M. Webb G. I. Zhang Y. Whisstock J. C. (2015). GlycoMine: a machine learning-based approach for predicting N-, C- and O-linked glycosylation in the human proteome. *Bioinformatics* 31 1411–1419. 10.1093/bioinformatics/btu852 25568279

[B37] Li S. H. Zhang J. Zhao Y. W. Dad F. Y. Ding H. Chen W. (2019). iPhoPred: a predictor for identifying phosphorylation sites in human protein. *IEEE Access.* 7 177517–177528. 10.1109/access.2019.2953951

[B38] Liao Z. J. Li D. P. Wang X. R. Li L. S. Zou Q. (2018). Cancer diagnosis through IsomiR expression with machine learning method. *Curr. Bioinform.* 13 57–63. 10.2174/1574893611666160609081155

[B39] Liu B. Han L. Liu X. Wu J. Ma Q. (2019). Computational prediction of Sigma-54 promoters in bacterial genomes by integrating motif finding and machine learning strategies. *IEEE/ACM Trans. Comput. Biol. Bioinform.* 16 1211–1218. 10.1109/tcbb.2018.2816032 29993815

[B40] Liu K. Chen W. (2020). iMRM: a platform for simultaneously identifying multiple kinds of RNA modifications. *Bioinformatics* 36 3336–3342. 10.1093/bioinformatics/btaa155 32134472

[B41] Liu Y. Guo J. Hu G. Zhu H. (2013). Gene prediction in metagenomic fragments based on the SVM algorithm. *BMC Bioinformatics* 14(Suppl. 5):S12. 10.1186/1471-2105-14-S5-S12 23735199 PMC3622649

[B42] Lu Y. Li C. Chen H. Zhong W. (2018). Identification of hub genes and analysis of prognostic values in pancreatic ductal adenocarcinoma by integrated bioinformatics methods. *Mol. Biol. Rep.* 45 1799–1807. 10.1007/s11033-018-4325-2 30173393

[B43] Manavalan B. Basith S. Shin T. H. Wei L. Lee G. (2019a). AtbPpred: a robust sequence-based prediction of anti-tubercular peptides using extremely randomized trees. *Comput. Struct. Biotechnol. J.* 17 972–981. 10.1016/j.csbj.2019.06.024 31372196 PMC6658830

[B44] Manavalan B. Basith S. Shin T. H. Wei L. Y. Lee G. (2019b). Meta-4mCpred: a sequence-based meta-predictor for accurate DNA 4mC site prediction using effective feature representation. *Mol. Ther-Nucl Acids* 16 733–744. 10.1016/j.omtn.2019.04.019 31146255 PMC6540332

[B45] Nguyen Kovochich A. Arensman M. Lay A. R. Rao N. P. Donahue T. Li X. (2013). HOXB7 promotes invasion and predicts survival in pancreatic adenocarcinoma. *Cancer* 119 529–539. 10.1002/cncr.27725 22914903 PMC3867310

[B46] Pan Z. Li L. Fang Q. Zhang Y. Hu X. Qian Y. (2018). Analysis of dynamic molecular networks for pancreatic ductal adenocarcinoma progression. *Cancer cell international* 18:214. 10.1186/s12935-018-0718-5 PMC6303882 30598639

[B47] Patil K. Chouhan U. (2019). Relevance of machine learning techniques and various protein features in protein fold classification: a review. *Curr. Bioinform.* 14 688–697. 10.2174/1574893614666190204154038

[B48] Peng H. Long F. Ding C. (2005). Feature selection based on mutual information: criteria of max-dependency, max-relevance, and min-redundancy. *IEEE Trans. Pattern Anal. Mach. Intell.* 27 1226–1238. 10.1109/tpami.2005.159 16119262

[B49] Schaduangrat N. Nantasenamat C. Prachayasittikul V. Shoombuatong W. (2019). ACPred: a computational tool for the prediction and analysis of anticancer peptides. *Molecules* 24:1973. 10.3390/molecules24101973 31121946 PMC6571645

[B50] Shoombuatong W. Hongjaisee S. Barin F. Chaijaruwanich J. Samleerat T. (2012). HIV-1 CRF01_AE coreceptor usage prediction using kernel methods based logistic model trees. *Comput. Biol. Med.* 42 885–889. 10.1016/j.compbiomed.2012.06.011 22824642

[B51] Song J. Tan H. Mahmood K. Law R. H. Buckle A. M. Webb G. I. (2009). Prodepth: predict residue depth by support vector regression approach from protein sequences only. *PLoS One* 4:e7072. 10.1371/journal.pone.0007072 19759917 PMC2742725

[B52] Stephenson N. Shane E. Chase J. Rowland J. Ries D. Justice N. (2019). Survey of machine learning techniques in drug discovery. *Curr. Drug Metab* 20 185–193. 10.2174/1389200219666180820112457 30124147

[B53] Tanaka S. (2016). Molecular pathogenesis and targeted therapy of pancreatic cancer. *Ann. Surg. Oncol.* 23(Suppl. 2), S197–S205. 25749932 10.1245/s10434-015-4463-x

[B54] Tang H. Cao R. Z. Wang W. Liu T. S. Wang L. M. He C. M. (2017). A two-step discriminated method to identify thermophilic proteins. *Int. J. Biomath.* 10:1750050 10.1142/s1793524517500504

[B55] Tripathi S. Pohl M. O. Zhou Y. Rodriguez-Frandsen A. Wang G. Stein D. A. (2015). Meta- and orthogonal integration of influenza “OMICs” data defines a role for UBR4 in virus budding. *Cell Host Microbe* 18 723–735. 10.1016/j.chom.2015.11.002 26651948 PMC4829074

[B56] Vaschetto L. M. (2019). The emergence of non-coding RNAs as versatile and efficient therapeutic tools. *Curr. Gene Ther.* 19 289–289. 10.2174/156652321905191122154955 31889480

[B57] Wang X. Yin L. Yang L. Zheng Y. Liu S. Yang J. (2019). Silencing ubiquitin-conjugating enzyme 2C inhibits proliferation and epithelial-mesenchymal transition in pancreatic ductal adenocarcinoma. *FEBS J.* 286 4889–4909. 10.1111/febs.15134 31715067

[B58] Win T. S. Malik A. A. Prachayasittikul V. Wikberg J. E. S. Nantasenamat C. Shoombuatong W. (2017). HemoPred: a web server for predicting the hemolytic activity of peptides. *Future Med. Chem.* 9 275–291. 10.4155/fmc-2016-0188 28211294

[B59] Win T. S. Schaduangrat N. Prachayasittikul V. Nantasenamat C. Shoombuatong W. (2018). PAAP: a web server for predicting antihypertensive activity of peptides. *Future Med. Chem.* 10 1749–1767. 10.4155/fmc-2017-0300 30039980

[B60] Wu Y. G. Lu X. X. Shen B. Zeng Y. (2019). The therapeutic potential and role of miRNA, lncRNA, and circRNA in osteoarthritis. *Curr. Gene Ther.* 19 255–263. 10.2174/1566523219666190716092203 31333128

[B61] Yan H. Li M. Cao L. (2019). A robust qualitative transcriptional signature for the correct pathological diagnosis of gastric cancer. *J. Trans. Med.* 17:63. 10.1186/s12967-019-1816-4 PMC6394047 30819200

[B62] Yang C. Liu Z. Zeng X. Wu Q. Liao X. Wang X. (2018). Evaluation of the diagnostic ability of laminin gene family for pancreatic ductal adenocarcinoma. *Aging* 11 3679–3703. 10.18632/aging.102007 31182680 PMC6594799

[B63] Zhang R. Leng H. Huang J. Du Y. Wang Y. Zang W. (2014). miR-337 regulates the proliferation and invasion in pancreatic ductal adenocarcinoma by targeting HOXB7. *Diagnostic Pathol.* 9:171. 10.1186/s13000-014-0171-2 PMC4164712 25183455

[B64] Zhang Y. Zoltan M. Riquelme E. Xu H. Sahin I. Castro-Pando S. (2018a). Immune cell production of interleukin 17 induces stem cell features of pancreatic intraepithelial neoplasia cells. *Gastroenterology* 155 210–223.e3. 29604293 10.1053/j.gastro.2018.03.041 PMC6035075

[B65] Zhang Z. Pan B. Lv S. Ji Z. Wu Q. Lang R. (2018b). Integrating MicroRNA expression profiling studies to systematically evaluate the diagnostic value of MicroRNAs in pancreatic cancer and validate their prognostic significance with the cancer genome atlas data. *Cell. Physiol. Biochem.* 49 678–695. 10.1159/000493033 30165365

[B66] Zhao W. Chen B. Guo X. Wang R. Chang Z. Dong Y. (2016). A rank-based transcriptional signature for predicting relapse risk of stage II colorectal cancer identified with proper data sources. *Oncotarget* 7 19060–19071. 10.18632/oncotarget.7956 26967049 PMC4951352

[B67] Zou Q. Ma Q. (2020). The application of machine learning to disease diagnosis and treatment. *Math. Biosci.* 320:108305. 10.1016/j.mbs.2019.108305 31857093

[B68] Zou Q. Qu K. Luo Y. Yin D. Ju Y. Tang H. (2018). Predicting diabetes mellitus with machine learning techniques. *Front. Genet.* 9:515. 10.3389/fgene.2018.00515 30459809 PMC6232260

